# Reverse takotsubo cardiomyopathy in fulminant COVID-19 associated with cytokine release syndrome and resolution following therapeutic plasma exchange: a case-report

**DOI:** 10.1186/s12872-020-01665-0

**Published:** 2020-08-26

**Authors:** Fahad Faqihi, Abdulrahman Alharthy, Rayan Alshaya, John Papanikolaou, Demetrios J. Kutsogiannis, Peter G. Brindley, Dimitrios Karakitsos

**Affiliations:** 1 Critical Care Department, King Saud Medical City, Riyadh, Kingdom of Saudi Arabia; 2 Critical Care Department, Al Imam Abdulrahman Al Feisal Hospital, Riyadh, Kingdom of Saudi Arabia; 3 Department of Medicine, School of Medicine, University of South Carolina, Columbia, SC USA; 4 Department of Critical Care, Faculty of Medicine and Dentistry, The University of Alberta, Edmonton, AB Canada; 5 Critical Care Department, Keck Medical School, USC, Los Angeles, CA USA

**Keywords:** COVID-19, Reverse takotsubo cardiomyopathy, Echocardiography, Cardiogenic shock, Cytokine release syndrome, Therapeutic plasma exchange, Case-report

## Abstract

**Background:**

Fulminant (life-threatening) COVID-19 can be associated with acute respiratory failure (ARF), multi-system organ failure and cytokine release syndrome (CRS). We present a rare case of fulminant COVID-19 associated with reverse-takotsubo-cardiomyopathy (RTCC) that improved with therapeutic plasma exchange (TPE).

**Case presentation:**

A 40 year old previous healthy male presented in the emergency room with 4 days of dry cough, chest pain, myalgias and fatigue. He progressed to ARF requiring high-flow-nasal-cannula (flow: 60 L/minute, fraction of inspired oxygen: 40%). Real-Time-Polymerase-Chain-Reaction (RT-PCR) assay confirmed COVID-19 and chest X-ray showed interstitial infiltrates. Biochemistry suggested CRS: increased C-reactive protein, lactate dehydrogenase, ferritin and interleukin-6. Renal function was normal but lactate levels were elevated. Electrocardiogram demonstrated non-specific changes and troponin-I levels were slightly elevated. Echocardiography revealed left ventricular (LV) basal and midventricular akinesia with apex sparing (LV ejection fraction: 30%) and depressed cardiac output (2.8 L/min) consistent with a rare variant of stress-related cardiomyopathy: RTCC. His ratio of partial arterial pressure of oxygen to fractional inspired concentration of oxygen was < 120. He was admitted to the intensive care unit (ICU) for mechanical ventilation and vasopressors, plus antivirals (lopinavir/ritonavir), and prophylactic anticoagulation. Infusion of milrinone failed to improve his cardiogenic shock (day-1). Thus, rescue TPE was performed using the Spectra Optia™ Apheresis System equipped with the Depuro D2000 Adsorption Cartridge (Terumo BCT Inc., USA) without protective antibodies. Over 5 days he received daily TPE (each lasting 4 hours). His lactate levels, oxygenation, and LV function normalized and he was weaned off vasopressors. His inflammation markers improved, and he was extubated on day-7. RT-PCR was negative on day-17. He was discharged to home isolation in good condition.

**Conclusion:**

Stress-cardiomyopathy may complicate the course of fulminant COVID-19 with associated CRS. If inotropic therapy fails, TPE without protective antibodies may help rescue the critically ill patient.

## Background

The novel coronavirus SARS-CoV-2 disease (COVID-19) has emerged in Wuhan city, Hubei province, in China, and has spread worldwide [1]. A minority of patients can develop life-threatening disease, which is characterized by acute respiratory distress syndrome (ARDS), sepsis, multi-system organ failure (MSOF), thromboembolic disease, neurological manifestations, and associated cytokine release syndrome (CRS) [2, 3]. Cardiac involvement in COVID-19 includes arrhythmia (atrial fibrillation, ventricular tachyarrhythmia and fibrillation), cardiac injury [elevated troponin I and creatine kinase (CK) levels], fulminant myocarditis, heart failure, and pulmonary embolism (PE) [4–9]. The etiology is assumed multifactorial, including direct viral myocardial injury, hypoxia, hypotension, ACE2-receptor down-regulation, drug toxicity, CRS, and endogenous catecholamine over-discharge [4–9]. Of note, the histopathologic findings in autopsies of COVID-19 patients have not definitively confirmed myocarditis [10–12].

Recent studies have suggested the occurrence of takotsubo cardiomyopathy (TTC) [13–17], and reverse takotsubo cardiomyopathy (RTCC) [18] in patients with COVID-19. The underlying pathophysiology of TTC (or neurogenic stress cardiomyopathy) is believed multifactorial with catecholamine-mediated cardiotoxicity the most likely mechanism [19–24]. Although usually reversible [25–28], TCC can cause severe left ventricular (LV) wall dysfunction (with an echocardiographic picture of apical ballooning), acute heart failure, cardiogenic shock, LV outflow tract obstruction, thrombus formation, and arrhythmias [29]. Reverse TTC (RTTC) is a rare variant, which is characterized by basal and midventricular LV akinesia plus apical sparing [18, 27]. Herein, we present a patient with life-threatening COVID-19 who had ARDS, CRS and RTTC [30]. These resolved with therapeutic plasma exchange (TPE) without protective antibodies. TPE has been previously used in patients with severe sepsis, MSOF and SARS-CoV with variable results [31–35].

## Case presentation

**Fig. 1:**
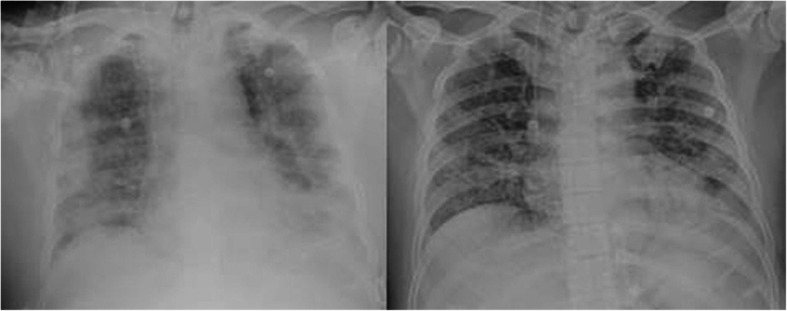
Portable chest X-rays of our COVID-19 patient depicting interstitial infiltrates and consolidations (day-1) prior to therapeutic plasma exchange (TPE) (*left panel*); and after five sessions of TPE (day-5) showing gradual improvement (*right panel*)

**Fig. 2:**
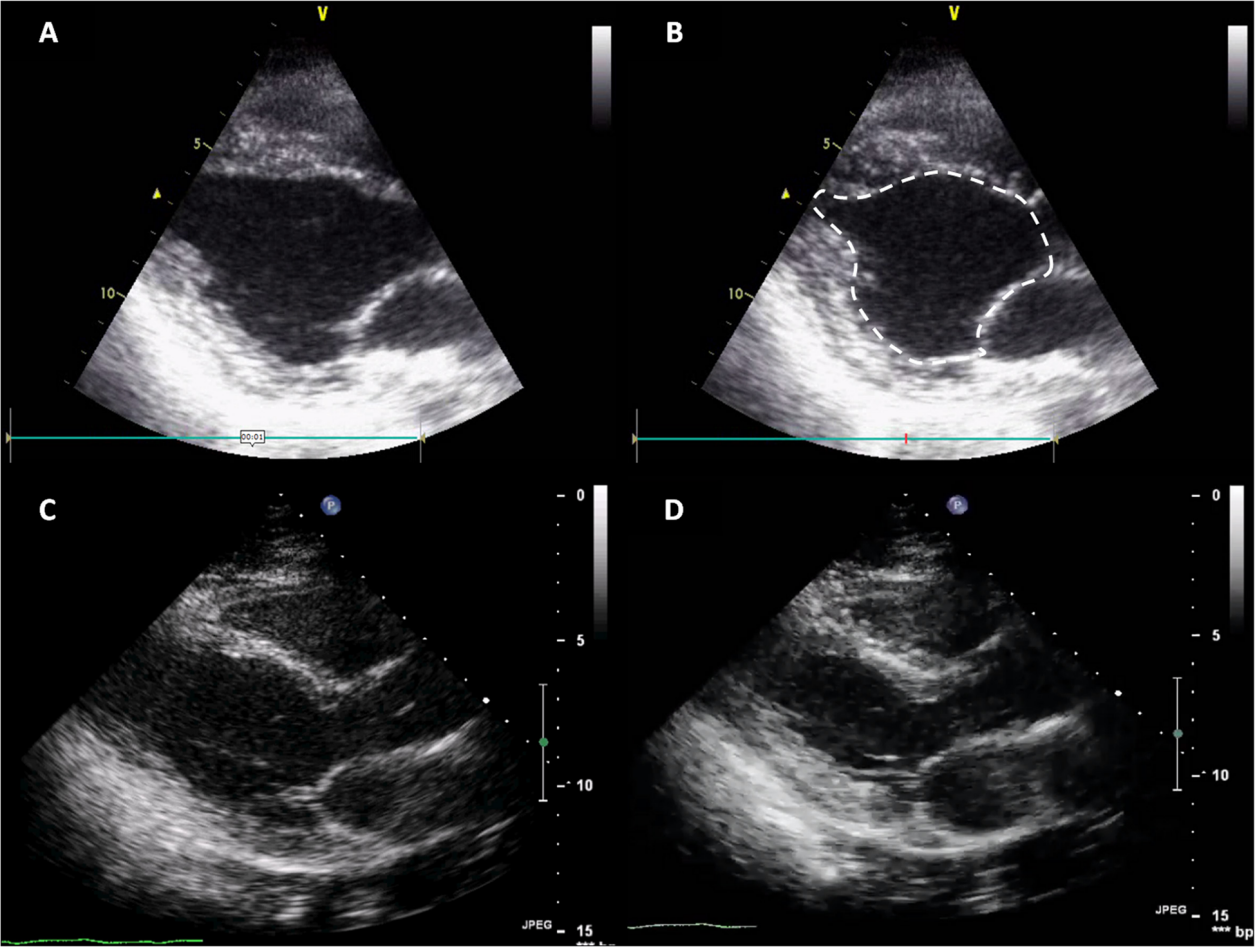
Τwo-dimensional bedside echocardiography (parasternal long-axis views) depicting the anteroseptal and posterolateral walls of the left ventricle (LV) in our COVID-19 patient (day-1). End-diastolic (**a**) and end-systolic (**b**) frames illustrating basal and midventricular LV akinesia with apical sparing and an “ace of spades” configuration typical of reverse takotsubo cardiomyopathy (*white dashed line; frame B*). End-diastolic (**c**) and end-systolic (**d**) frames after two plasma exchange sessions (day-2) showing gradual recovery of the segmental LV wall motion abnormalities


A 40 year old, previous healthy, male presented to the emergency department with 4 days of dry cough, chest pain, myalgia and progressive fatigue. Physical examination was normal apart from decreased breath sounds at the lung bases and mild tachypnea. Vital signs were within normal limits. Within 2 hours, however, the patient desaturated (Pulse Oximeter Oxygen Saturation: 86%) and became dyspneic; hence he received high flow nasal cannula (HFNC; flow: 60 L/minute, fraction of inspired oxygen 40%) [36–38]. Laboratory results were within normal limits apart from lymphocytopenia (0.55 × 10^9^/L, normal: 1.1–3.2 × 10^9^/L), and increased C-reactive protein [(CRP) 82.5 mg/liter, normal: 0–7 mg/liter], lactate dehydrogenase [(LDH) 840 units/liter, normal: 100 to 190 units/liter], ferritin (3101 ng/ml, normal: 23–336 ng/ml), and interleukin-6 [(IL-6) 398 pg/ml, normal: 1–7 pg/mL). The increased values of the inflammation biomarkers laboratory suggested the development of CRS, which is further defined in Table 1 (Additional file 1) [39, 40]. CK was slightly increased (422 units/liter, normal: 22 to 198 units/liter) but renal function was normal. D-dimer levels were normal but troponin-I was slightly elevated (4.7 ng/ml, normal: < 0.04 ng/ml). Electrocardiogram showed sinus tachycardia (115 beats/min) and non-specific ST-segment and T-wave abnormalities in the precordial leads. Nasopharyngeal swabs confirmed COVID-19 by Real-Time-Polymerase-Chain-Reaction (RT-PCR) assays (Roche, Basel, Switzerland) [41, 42]. Chest X-ray showed interstitial infiltrates and consolidations (Fig. 1). Transthoracic two-dimensional echocardiography revealed LV basal and midventricular akinesia with apical sparing (Fig. 2). LV ejection fraction was approximately 30% and cardiac output was depressed (2.8 L/min) [43–45]. The slightly elevated troponin I levels and the echocardiographic findings suggested RTCC [13–18, 25–28]. The right ventricle was not significantly dilated, and no LV outflow tract obstruction, thrombus or pericardial effusion was evident. Despite the prompt application of HFNC the patient continued to deteriorate due to cardiogenic shock (Video 1; Additional files 1 and 2), with increasing noradrenaline requirements (1.5 mcg/kg/ min) and serum lactate (5.6 mmol/L). We could not perform computed tomography angiography/coronary angiography due to his instability; hence, we could not definitively exclude pathologies such as aortic dissection, pulmonary embolism (PE), myocarditis or coronary artery disease (CAD). However, these diagnoses were less likely given the echocardiographic features of RTCC.

**Additional file 2.**


The patient was subsequently intubated and admitted to the intensive care unit (ICU), where his ratio of partial arterial pressure of oxygen to fractional inspired concentration of oxygen (PaO_2_/FiO_2_) was < 120. He received ARDS-net ventilation, lung recruitment, prone positioning, empiric antiviral therapy (lopinavir /ritonavir: 400/100 mg twice daily for 2 weeks) [46], and prophylactic anticoagulation (enoxaparin plus intermittent pneumatic compression) [10, 47–50]. Upon ICU admission (day-1), he required increasing norepinephrine (1.8 mcg/kg/min) due to worsening cardiogenic shock. Dobutamine was considered but held as this exogenous catecholamine-inotrope can aggravate stress-cardiomyopathy [13–18]. Levosimendan, a non-catecholamine inotrope, which shows promise in TCC, was not available [51]. Instead, intravenous milrinone (non-catecholamine inotrope) was initiated: 50 mcg/kg loading dose over 10 min; then 0.375mcg/kg/min. It was stopped due to tachyarrhythmia [52]. Intravenous beta-blocker esmolol (0.02 mg/kg /min) was titrated to a heart-rate ≤ 95 beats/min (to protect the heart from catecholamine storm and counteracting tachycardia) [53], but was stopped due to bronchospasm.

Because inotropic therapy was failing, we recruited the patient into our study investigating the potential role of TPE as rescue therapy in life-threatening COVID-19 associated with CRS [40]. Following informed consent from his legal representative, rescue TPE was initiated on post-ICU admission day-1, using the Spectra Optia™ Apheresis System equipped with the Depuro D2000 Adsorption Cartridge (Terumo BCT Inc., USA) [32, 33]. A dose of 1.5 plasma volumes was used for the first dose then one plasma volume daily for a total of five doses (4 hours each day). Spectra Optia™ Apheresis System employs an acid-citrate dextrose anticoagulant as per Kidney Disease Improving Global Outcomes 2019 guidelines [54]. Intravenous hydrocortisone 100 mg and chlorpheniramine 10 mg were administered as adjunctive treatment during TPE to reduce any potential side-effects. Plasma was replaced with albumin 5%. Our patient did not have any coagulopathy or elevated levels of D-dimer. No side effects due to TPE were recorded (i.e., allergies, infection, and coagulopathy). Daily negative fluid balance was achieved.

After only the 2nd TPE session (day-2) LV function gradually improved (Fig. 2) although interstitial lung edema was still present (Video 2; Additional files 1 and 3). After the 3rd TPE session (day-3), he was weaned off vasopressors, and his lactate, blood pressure and LV function normalized. After completion of TPE (day-5), PaO_2_/FiO_2_ ratio exceeded 300, his chest X-ray showed less infiltrates (Fig. 1), his lymphocyte count increased (from 0.55 to 1.1 × 10^9^/l) and his CRP (82.5 to 10.1 mg/liter), LDH (840 to 140 units/liter), ferritin (3101 to 234 ng/ml) and IL-6 (398 to 11 pg/ml) decreased. He was extubated on day-7 post-ICU admission. RT-PCR test and microbiology were negative on day-17. All work-up for systemic and other viral diseases was negative. The patient was pleased with his therapy but refused any additional diagnostic testing. He was discharged to home isolation in good condition, and has been followed by our outreach team for a period of 2 months.

## Discussion and conclusions

This rare case of RTCC in COVID-19 comes from our ongoing study of TPE as rescue therapy in life threatening COVID-19 with associated CRS (ISRCTN21363594) [40]. TTC may present as severe LV systolic dysfunction after emotional stress (primary subtype) or critical illness (secondary subtype). Although usually transient, TTC can manifest as cardiogenic shock and be associated with lower short- and long- term survival [13–29]. Transient stress-cardiomyopathy has been reported in COVID-19 [13–18]. An overlapping picture of myocarditis and RTCC has been reported too [18]. Our patient had typical clinical and echocardiographic features of RTCC [13–29]. We could not perform a full diagnostic work-up due to the severity of COVID-19; hence, we could not definitively exclude other cardiovascular pathologies such as aortic dissection, PE, myocarditis or CAD. However, his echocardiographic features and biochemical abnormalities make these diagnoses less likely.

Importantly, we cannot conclude that TPE definitively rescued the patient. Potentially, his clinical state and biochemical aberrations could have improved without TPE; although his clinical picture was grave, and other therapies largely failed. The management of acute heart failure due to TTC, especially in patients with life-threatening COVID-19, is challenging. Patients with stress-cardiomyopathy often do not improve with fluid administration because their LV operates mainly within the flat portion of the Frank-Starling curve. This means aggressive volume resuscitation can cause pulmonary edema and hemodynamic compromise. In TCC, adrenergic stimulation is high, thus exogenous catecholamine-inotropes (i.e., dopamine, dobutamine, and norepinephrine) can further impair cardiac contractility: by exacerbating neurocardiogenic injury and calcium overload in an already stressed myocardium. The use of non-catecholamine inotropes (i.e., milrinone and levosimendan) may be beneficial providing no LV outflow obstruction [13–18, 51–53]. Beta-blockers are important in the management of TTC complicated by LV outflow tract obstruction [55]; however, their use in RTTC without intraventricular pressure gradient remains controversial [56]. Our patient did not have LV outflow tract obstruction; however, using the beta-1 cardio-selective blocker esmolol (half-life: 9 min) has been encouraged in TTC. Fortunately this short acting agent can be discontinued if adverse effects occur (i.e., impaired LV cardiac contractility, blood pressure, and central hemodynamics) [57]. Recently, an in-hospital score has been suggested to stratify TTS patient’s risk by the German and Italian Stress Cardiomyopathy (GEIST) registry [58]. That study concluded that independent predictors of in-hospital complications of TTC were: male sex, history of neurologic disorder, RV involvement, and decreased LVEF (GEIST score). Hence, our patient would be at intermediate risk being a male with an LVEF of 30%, and requiring ICU admission. Notwithstanding, our patient also had life-threatening COVID-19, which might be considered an additional risk factor complicating acute heart failure due to stress-cardiomyopathy.

This is the first time, to our knowledge, that TPE has been reported as rescue therapy for cardiogenic shock due to stress-cardiomyopathy in serious COVID-19. We employed TPE with an adsorption cartridge containing activated uncoated coconut shell (carbon granules) charcoal (100 g), and the nonionic resins Amberlite XAD-7HP and Amberchrom GC300C [20, 28]. These can remove interferon-gamma, interleukins − 3, − 10, −1B, − 6, − 8, and tumor necrosis factor-alpha [18–23]. Although a full cytokine panel was not available in our institution at that time, TPE resulted in pertinent biochemical improvements: decreased inflammatory mediators (CRP, LDH, and ferritin), IL-6, and increased lymphocytic count [30–40]. Elevated inflammation markers are associated with more severe COVID-19, and elevated IL-6 is associated with worse CRS [30–40]. This is why tocilizumab, a monoclonal antibody against IL-6, has been tried in severe COVID-19, albeit with conflicting results [59–62]. In our institution, tocilizumab was not available, at that time. Similarly, the antiviral remdesevir and convalescent plasma transfusion (containing a high concentration of neutralizing antibodies) have shown promise in treating serious COVID-19 but results are inconclusive [63–66]. In a recent, randomized control trial, which has been performed among patients with severe or life-threatening COVID-19, convalescent plasma therapy has been added to standard anti-COVID-19 treatment. However, when compared with standard treatment alone, convalescent plasma therapy was not associated with a significant clinical improvement within 28 days of hospitalization [67]. Moreover, the natural course of COVID-19 viremia and the developing antibodies titers remains uncertain [68–70], and convalescent plasma therapy is not currently widely available.

This case-report has limitations, which prevent its generalizability. First, patients with cardiogenic shock and absence of LV outflow obstruction might benefit from an intra-aortic balloon pump, but this was not available [71]. Also, cardiac magnetic resonance imaging was not performed to exclude myocarditis [14–18] as our follow-up period was limited to 2 months. Importantly, RTCC (as well as COVID-19) can resolve with no more than supportive therapy. In other words we cannot definitively confirm that TPE rescued the patient, only that their illness severity led us to apply TPE, and the patient survived. Other limitations include that in trying to pinpoint the role played by TPE we cannot exclude other aggressive life support plus empiric therapies. The effects of all of these on biochemistry, cardiac function and survival is unclear [1, 2, 4–18, 72]. Even if TPE is beneficial, the optimal TPE regime is unclear because the course of COVID-19 viremia has yet to be elucidated [63–68]. Accordingly, we speculate that prompt TPE initiation mitigated full-blown CRS, given the associated reduction in our patient’s elevated IL-6 [30–35, 59–62]. Presumably, at an early stage of COVID-19 dysregulated immune system pathology may be more important than viral replication per se [73]. However, it is unknown exactly how reducing COVID-19 associated CRS works in improving stress-cardiomyopathy. It could have a direct effect (i.e., by reducing cardiac inflammation) or work indirectly (i.e., improving oxygenation, LV filling pressures and reducing the stress of critical illness).

In conclusion, stress-cardiomyopathy and related cardiogenic shock can complicate fulminant COVID-19 with associated CRS. If usual therapies fail, rescue TPE (without protective antibodies) could be considered and warrants further study.

## Supplementary information

**Additional file 1.**

**Additional file 3.**

**Additional file 4.**

## Data Availability

The datasets used and/or analyzed during the current study are available from the corresponding author on reasonable request.

## Abbreviations

TCC: Takotsubo cardiomyopathy
RTCC: Reverse takotsubo cardiomyopathy
COVID-19: SARS-CoV-2 disease
TPE: Therapeutic plasma exchange
ICU: Intensive care unit
CRS: Cytokine release syndrome
ARDS: Acute respiratory distress syndrome
MSOF: Multi-system organ failure
CRP: C-reactive protein
LDH: Lactate dehydrogenase
IL-6: Interleukin-6
LV: Left ventricular
RT-PCR: Real-time-polymerase-chain-reaction
HFNC: High flow nasal cannula
PaO_2_/FiO_2_ ratio: Partial arterial pressure of oxygen to fractional inspired concentration of oxygen ratio
PE: Pulmonary embolism
CAD: Coronary artery disease
