# Supplementary material for: Reverse takotsubo cardiomyopathy in fulminant COVID-19 associated with cytokine release syndrome and resolution following therapeutic plasma exchange: a case-report
Source: BMC Cardiovasc Disord. 2020 Aug 26;20:389. doi: 10.1186/s12872-020-01665-0 (PMC7447602; doi:10.1186/s12872-020-01665-0)
Supplement: Supplementary file 2 — Additional file 1. [file 12872_2020_1665_MOESM1_ESM.docx]

**Additional file 1**

**Table 1**. Criteria for the development of CRS in COVID-19.

| One or more of the following criteria should be present* |
| --- |
| C-reactive protein > 100 or > 50 mg/L but doubled in the past 48 hours |
| lymphocyte count < 0.6 x 10^9^/L |
| Serum Interleukin-6 (IL-6) ≥ 3x upper normal limit |
| Ferritin > 300 ug/L (or surrogate) with doubling within 24 hours |
| Ferritin > 600 ug/L at presentation and LDH >250 U/L |
| Elevated D-dimer (> 1 mcg/mL) |
| Abbreviations: CRS = cytokine release syndrome, LDH = lactate dehydrogenase; * We defined as low risk for developing CRS the presence of one criterion, moderate risk the presence of two to three criteria and high risk the presence of more than three criteria. |

**Video Legends Video 1.** Two-dimensional bedside echocardiography (parasternal long-axis views) showing basal and midventricular left ventricular (LV) akinesia with apical sparing, an “ace of spades” configuration and decreased LV ejection fraction typical of reverse takotsubo cardiomyopathy in our COVID-19 patient prior to intubation (day-1).  **Video 2.** Τwo-dimensional bedside echocardiography (parasternal long-axis views), after two plasma exchange sessions in our mechanically ventilated COVID-19 patient (day-2), showing gradual improvement of left ventricular function; while, interstitial lung edema still exists [please, note the B-lines (vertical “beam lines” originating from the pleural line) overlaying the cardiac image during positive inspiratory pressures].
